# Diagnostic value of Tc-99m-MIBI SPECT/CT in parathyroid carcinoma with lung metastasis: a case report and literature review

**DOI:** 10.3389/fonc.2024.1501447

**Published:** 2024-12-24

**Authors:** Yirong Zhu, Lian Wang, Jiaxi You, Shengming Deng, Yizhen Shi, Zengli Liu, Zhihui Hong

**Affiliations:** ^1^ Department of Nuclear Medicine, The Second Affiliated Hospital of Soochow University, Suzhou, China; ^2^ Shanghai Key Laboratory of Molecular Imaging, Shanghai University of Medicine and Health Sciences, Shanghai, China; ^3^ Department of Oncology, Xuyi People’s Hospital, Xuyi, China; ^4^ Department of Nuclear Medicine, The First Affiliated Hospital of Soochow University, Suzhou, China; ^5^ State Key Laboratory of Radiation Medicine and Protection, Soochow University, Suzhou, China; ^6^ National Health Commission (NHC) Key Laboratory of Nuclear Medicine and Jiangsu Key Laboratory of Molecular Nuclear Medicine, Wuxi, China

**Keywords:** parathyroid carcinoma, lung metastasis, PTH, Tc-99m-MIBI SPECT/CT, diagnosis, case report

## Abstract

**Purpose:**

Parathyroid carcinoma (PC) is an extremely rare disease, typically presenting with marked elevations of serum calcium concentrations and associated with significantly increased parathyroid hormone (PTH) levels. Although it progresses slowly, approximately25% of PC patients have lung metastases. In the present study, we aimed to evaluate the role of technetium-99m methoxy isobutyl isonitrile (Tc-99m-MIBI; sestamibi) SPECT/CT scintigraphy in the preoperative localization of parathyroid adenomas, incidental metastases findings of PC, and ectopic parathyroid tissue.

**Methods:**

We presented a rare case alongside a review of the relevant literature.

**Results:**

We described an unusual case of a 25-year-old female patient with co-occurrence of PC and lung metastasis. The primary PC lesion showed no radioactive uptake, while the lung metastasis presented as a hyperfunctioning nodule, successfully localized using Tc-99m-MIBI SPECT/CT and confirmed through surgical and pathological examination.

**Conclusions:**

This case emphasized the uniqueness of Tc-99m-MIBI SPECT/CT imaging in diagnosing PC and metastatic lesions. The appropriate application of this technique may help avert the aggressive clinical progression of PC.

## Introduction

Primary hyperparathyroidism (PHPT) is a common cause of hypercalcemia, with parathyroid adenomas accounting for 80%-85% of cases, hyperplasia for 10-15%, and parathyroid carcinoma (PC)comprising less than 1% (1–3). PC is a highly aggressive endocrine tumor, with more than 90% of cases associated with excessive parathyroid hormone (PTH), and its annual incidence is estimated less than 1 case per million (4). Although recent developments in biochemical, molecular, and radiological techniques, PC remains an elusive disease to recognize clinically. Accurate diagnosis is difficult for various reasons, such as its rarity, the lack of specificity, and overlapping symptoms with parathyroid adenoma (PA). Therefore, most cases of PC are detected incidentally and confirmed by histopathology and characterized by vascular or capsular invasion (5).

Tc-99m-MIBI SPECT/CT, ultrasonography (US), X-ray computed tomography (CT) and magnetic resonance imaging (MRI) are recommended for accurate localization of parathyroid lesions prior to invasive parathyroidectomy. Among these, Tc-99m-MIBI and US of the neck are the most commonly utilized imaging modalities in clinical practice. However, Tc-99m-MIBI SPECT/CT plays an irreplaceable role in the accurate localization of parathyroid lesions, especially in detecting ectopic or metastatic lesions.

In the present study, we presented a rare case of nonfunctional PC with functional lung metastasis. While the primary parathyroid lesion exhibited no radioactive concentration, the lung metastasis showed a high concentration of radioactivity, was accurately localized through Tc-99m-MIBI SPECT/CT and confirmed by surgical and pathological examination. In the present study, we aimed to evaluate the characteristics of PC and assess the critical role of Tc-99m-MIBI SPECT/CT in localizing both primary PC and distant metastases. Moreover, the patient provided informed consent for publication of this case.

## Case presentation

A 25-year-old female presented with a 3-year history of episodic chest tightness and shortness of breath, which had worsened over the past 3 months. Initially, these symptoms appeared after emotional agitation, with episodes of chest discomfort and distress resolving with rest and without special treatment. In recent months, her condition deteriorated, manifesting as severe fatigue, low spirits, nausea, vomiting, anorexia, bone pain, and weight loss.

On August 18, 2017, she first consulted to the Gastroenterology Department of our hospital, and a gastroscopy revealed chronic gastritis. Three days later, she was referred to the Cardiology Department for evaluation due to a short Q-T interval identified on an electrocardiogram (ECG). For further evaluation and management, she was subsequently admitted with a preliminary diagnosis of “cardiomyopathy”.

On admission, her physical examinations revealed a heart rate of 100 beats/min and backache, with no other significant findings. Her medical history included a myomectomy performed in 2014, with no history of head or neck radiation exposure or a family history of thyroid or parathyroid cancer.

At the time of hospitalization, laboratory tests revealed severe hypercalcemia (5.37 mmol/L; normal range: 2.0-2.7 mmol/L) and a significantly increased serum PTH level (613.3 pg/mL; normal range: 12-88 pg/mL). Tumor marker assays and thyroid function tests were unremarkable. The patient was referred to the Endocrinology Department where she received calcium-lowering therapy.

**Figure 1:**
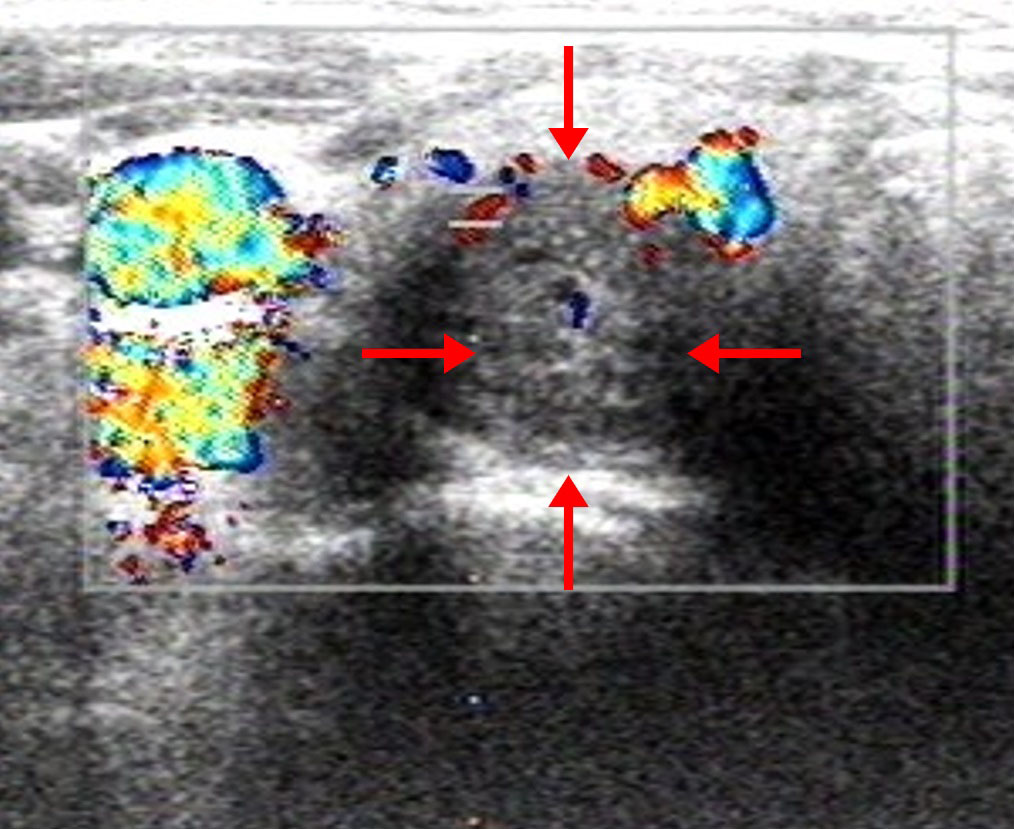
Parathyroid US of the patient. A 29×24×16 mm cystic- solid mixed-echo area in the posterior of the right lower thyroid gland with well-defined borders.

**Figure 2:**
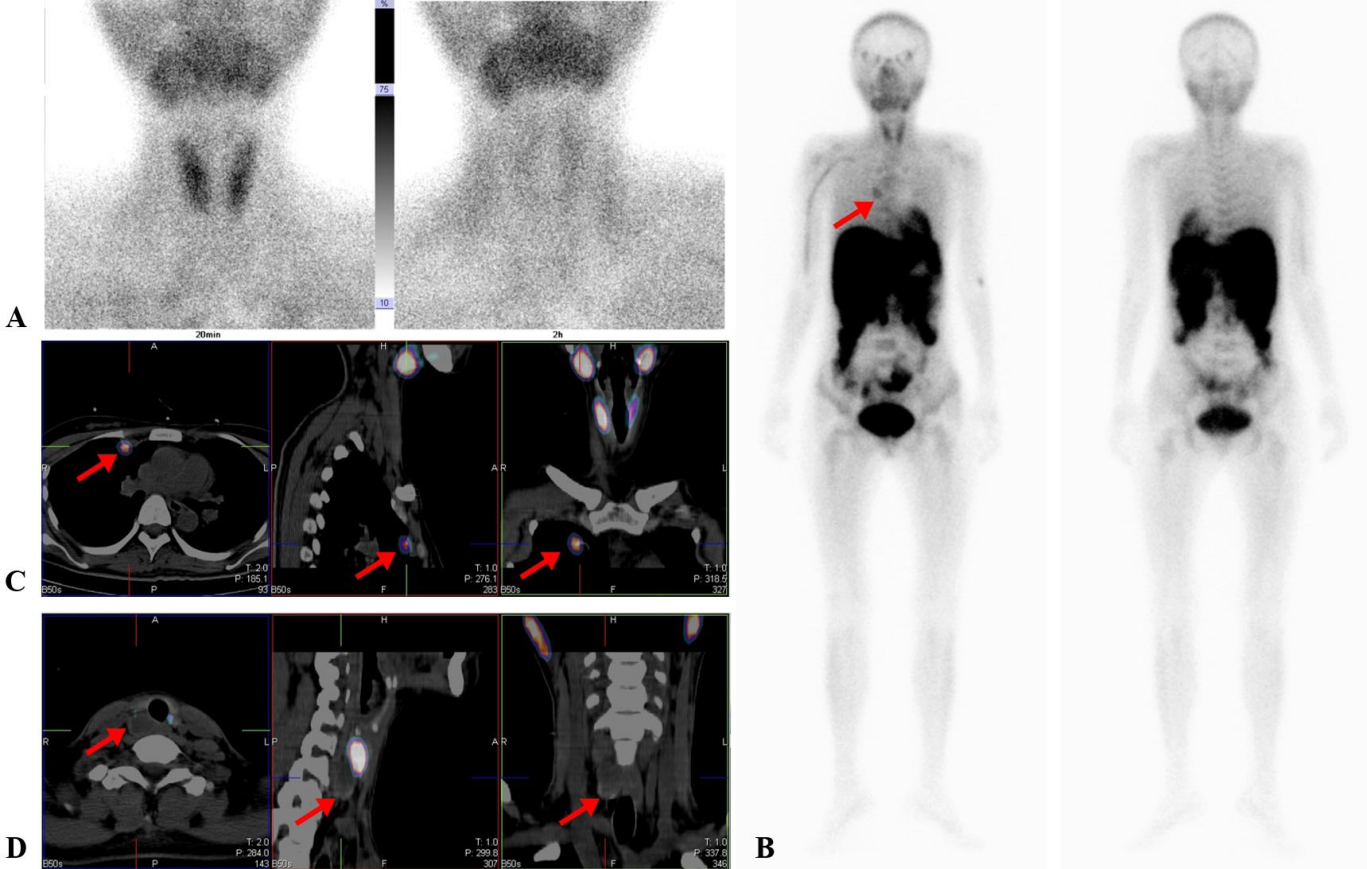
Dual-phase Tc-99m-sestamibi scintigraphy and SPECT/CT scans of the patient. **(A)** Early and delay imaging of dual-phase Tc-99m-sestamibi parathyroid scintigraphy. The thyroid gland was developed clearly in the early image and quickly decreased in the delayed image, whereas no radiotracer uptake was revealed at the lower pole of the right thyroid lobe both in early and delayed imaging. **(B)** Tc-99m-MIBI SPECT demonstrated a metastatic lesion in the right chest (arrow). **(C)** Tc-99m-MIBI SPECT/CT fusion images showed that high radiotracer uptake in the area of the right chest coincided with a mass in the upper right lung through CT scan (arrow). **(D)** Tc-99m-MIBI SPECT/CT fusion images showed that no survival of accumulation in the posterior of the right lower thyroid gland with low density (arrow).

An initial parathyroid ultrasound revealed no abnormalities, likely due to operator experience. A repeat US performed by an experienced radiologist identified an abnormal echo posterior to the right lower thyroid lobe, suspicious for parathyroid lesion (**Figure 1**). However, dual-phase Tc-99m-MIBI parathyroid scintigraphy failed to detect any parathyroid tumor on both early and delayed images (**Figure 2A**).

Subsequently, an imagological diagnosis was performed to localize potential hyperfunctioning parathyroid tissues. ATc-99m-MIBI whole-body scan (WBS) revealed a hyperfunctioning nodule in the right chest on both early and delayed images (**Figure 2B**). Delayed Tc-99m-MIBI SPECT/CT fusion images showed that the abnormal Tc-99m-MIBI uptake was located in the area of the right upper lung (**Figure 2C**), while the low-density lesion posterior to the right thyroid lobe showed no uptake (**Figure 2D**).

Based on clinical symptoms, laboratory results, and radiological image features, we initially suspected that the chest lesion was the metastasis of PC. However the possibility of an ectopic parathyroid gland could not be completely excluded at that stage.

The patient was subsequently referred to the Department of General Surgery, where she underwent a parathyroidectomy followed by a pneumonectomy. During the initial right parathyroidectomy, a 1.5×2.0 cm mass was identified posterior to the right thyroid gland. of the lesion displayed central areas of hemorrhage and necrosis. Subsequently, an operation of right lung wedge resection was carried out, disclosing a soft and friable mass about 2.0×3.0 cm near the horizontal fissure in the middle lobe of the right lung, densely adherent to the pleura.

Intraoperative parathyroid hormone assay showed a significant declined, although the level remained elevated at 112.5 pg/mL (normal range: 12-88 pg/mL).Notably, serum calcium normalized to 2.57 mmol/L (normal range: 2.0-2.7 mmol/L) 30 minutes post-operation.

**Figure 3:**
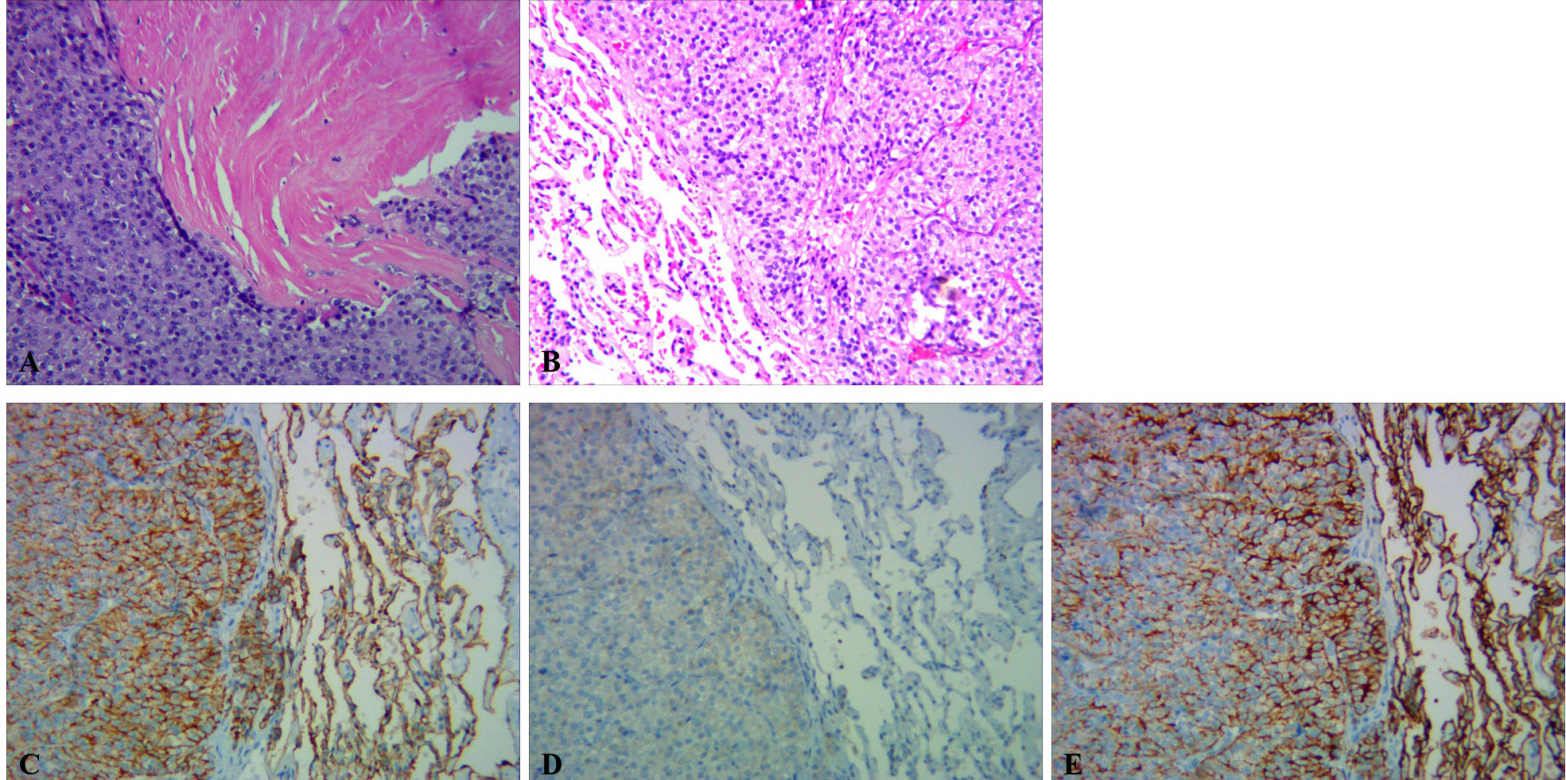
Histological sections of the PC and pulmonary lesion. **(A)** Histopathology (hematoxylin-eosin staining) of PC. The tumor had a solid growth pattern, which invaded the surrounding soft tissues with prominent necrosis (×100). **(B)** Histopathology (hematoxylin-eosin staining) of the pulmonary lesion. The pulmonary lesion was predominantly solid with round and atypical spindle stromal cells. **(C–E)** IHC of the pulmonary lesion staining revealed that the lesional cells were positive for AE1-AE3 **(C)**, Bcl2 **(D)**, and CK7 **(E)**, respectively (×100).

Histopathology and immunohistochemistry (IHC) results revealed that the cervical lesion in the posterior right thyroid lobe was PC, and the lung lesion was determined to be a metastasis site of the PC (**Figure 3**).

**Figure 4:**
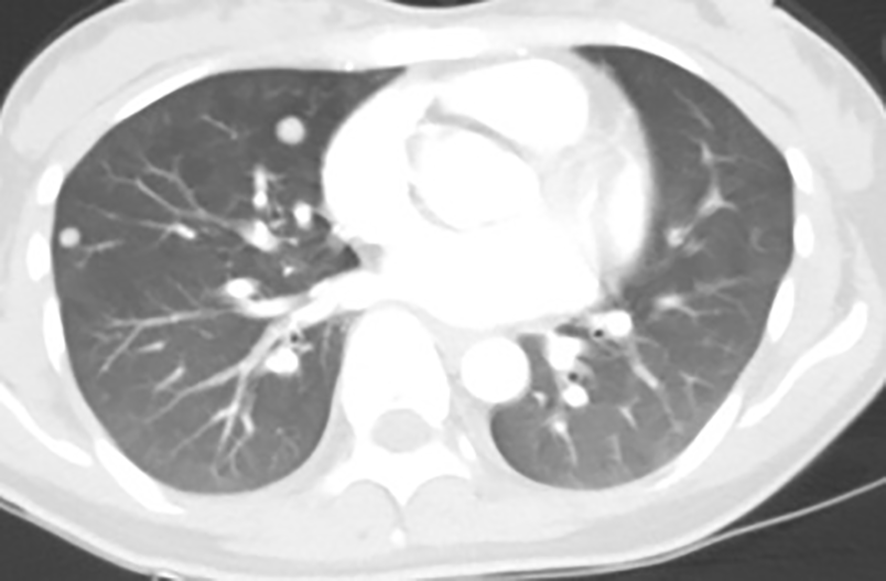
CT scan of the chest showed numbers of nodules with tissue density distributed on both lungs.

At one month post-operation, follow-up laboratory tests showed a persistently elevated PTH level of 323.8 pg/mL (normal range: 12-88 pg/mL), despite a normal serum calcium level of 2.15 mmol/L (normal range: 2.0-2.7 mmol/L). Neck and chest CT scans revealed the presence of metastatic nodules in both lungs (**Figure 4**).

## Discussion

PC was first described by De Quervain in 1904 (6). It occurs either as part of a genetic syndrome or sporadically, with an overall incidence of 3.5 to 5.7 per 10 million (7). Most PCs secrete high levels of PTH, leading tohypercalcemia associated with PHPT. Symptoms of hypercalcemia range from nonspecific manifestations, such as anorexia, nausea, vomiting, fatigue, and weakness, to more severe complications like polyuria, polydipsia, nephrocalcinosis, and renal failure. Moreover, other symptoms of hyperparathyroidism include bone pain, and fractures, which are similar to PA and difficult to differentiate.

Sharretts M et al. reported that PC patients were associated with serious hypercalcemia, high PTH level, and younger age, and these patients were prone to hypercalcemic crisis, renal dysfunction, and skeletal system changes (8). Current guideline of PC suggests that the possibility of PC should be considered in patients with greatly increased PTH level (>5-fold normal or >500 pg/mL) and severe hypercalcemia (>14 mg/dL) (4). In this case, the patients presented with a PTH level of 613.3 pg/mL and serum calcium of 96.66 mg/dL, strongly suggesting PC.

PC is a slow-growing disorder, with metastases occurring late, primarily in bones, lungs, and liver. The survival of PC patients with distant metastases is extremely low, especially for those with functioning distant metastases, which may also seriously affect the quality of life (9). Surgery remains the primary curative treatment for PC and its metastases, as radiotherapy and chemotherapy have limited role efficacy (4, 10).

In the present case, the patient underwent parathyroidectomy and pneumonectomy which initially reduced PTH and serum calcium levels significantly. However, the PTH level increased again at the one-month follow-up, despite normocalcemia. This suggested possible disease progression(recurrence or metastases), although ectopic parathyroid tissue could not be excluded. Subsequent neck and chest CT scans revealed the presence of metastatic nodules in both lungs.

Parathyroid carcinoma with metastases was associated with a high risk of local and distant recurrence, indicating a poor prognosis (11–14). Early and accurate localization of lesions is crucial for effective management. Non-invasive imaging modalities, such as Tc-99m-MIBI SPECT/CT, US, and CT, are routinely used to localize parathyroid lesions before invasive parathyroidectomy. US is a widely used, cost-effective, and sensitive method for diagnosing parathyroid lesions, but its accuracy depends heavily on the experience of the operator, and this modality has a suboptimal detection rate in silent areas, such as the mediastinum, tracheoesophageal groove, and retroesophageal region (15). CT offers better detection of lesions in the mediastinum but struggles to distinguish lymph nodes from parathyroid glands in the neck.

Tc-99m-MIBI parathyroid scintigraphy is particularly valuable for detecting parathyroid lesions with sensitivity rates of 50%-86% using the dual-phase protocol (16). However, false-negative results can occur due to factors such as o lesion size, cystic changes, hyperthyroidism, thyroiditis, or tumor composition (17). Additionally, up to 10% of PCs are non-functional, leading to false-negative results (8, 18, 19).

In cases where PTH and calcium levels are high but dual-phase Tc-99m-MIBI parathyroid scintigraphy is negative, a whole-body Tc-99m-MIBI (WBS) may be necessary to detect metastatic or ectopic lesions. Despite its high specificity and sensitivity, SPECT alone is limited by its inability to provide anatomical details. The hybrid SPECT/CT system overcomes this limitation by combining functional and anatomical imaging, resulting in improved diagnostic accuracy and image quality (20). **Table 1** summarized 11 cases of parathyroid carcinoma similar to this case reported in the past five years.

**Table 1:** Summary of parathyroid carcinoma case reports over the past five years.

Reference	Age/sex	PTH(pg/ml)	Ca(mmol/l)	PTH(pg/ml)	Ca(mmol/l)	Preoperative Dx	Pathology	Outcome
		Preoperative		Postoperative/X				
Elisa Dinoi, et al. (21)	53/M	581	14.9mg/dl	20/6M	9mg/dl	US: Posterior lower pole of the right thyroid MIBI: 7^th^ rib and the left scapula	Inferior Right PC; Scapula:brown tumor	MIBI: Bone Lesion Uptake Reduction (6-month follow-up)
Tianfeng Xu, et al. (22)	63/M	739	3.75	8.03/6M	2.35	US: Multiple nodular goiter; Left Mid-Lower Thyroid Lobe MIBI: Mild Uptake at posterior upper pole of the left thyroid	Left Upper PA; Right Intrathyroidal PC	No recurrence (6-month follow-up)
Suzune Tsukamoto, et al. (23)	69/F	1844	12.0 mg/dl	13.0/1D	8.9 mg/dl	^18^F-FDG: Uptake in the cervical mass and multiple osteolytic lesions MIBI: Uptake in the cervical mass; no uptake in the osteolytic lesions	Cervical mass:PC; Right clavicle: brown tumor	18F-FDG: Bone Lesion Uptake Reduction; No recurrence (6-month follow-up)
Jun Yang, et al. (24)	46/F	3356	3.22	63.6/1D	1.91	MIBI: Uptake in inferior pole of the left thyroid and right anterior cervical subcutaneous	Inferior Left PC; Right subcutaneous PA	PTH:190~320; Ca: 2.23–2.48 (8-month follow-up)
Yan Bao, et al. (25)	50/F	1562.3	4.63	19.2/1D	2.42	MIBI: High uptake in the mid-upper mediastinum	Mediastinal PC	No recurrence (6-month follow-up)
Ko Yokoyama, et al. (26)	54/F	1007	11.4 mg/dl	20/4M	8.7	US: Bilateral dorsal thyroid lobes MIBI: Uptake in superior pole of the left thyroid	Superior Left PC; Right PA	No recurrence (25-month follow-up)
Maxime Damien, et al. (27)	51/F	1600	4.66			MIBI: High uptake in the left thyroid lobe; Mild uptake in inferior pole of the right thyroid 11C-methionine and 18F-fluorocholine PET/CT: Hypermetabolism in superior pole of the left thyroid	Bifocal intrathyroidal PC	No follow-up
Wei Liu, et al. (28)	49/M	1483.1	2.59	49.6/N		MIBI: Uptake in the left and right thyroid upper poles, the left suprasternal fossa, and above the brachiocephalic trunk	Superior pole of left thyroid and the left suprasternal fossa PA; Superior pole of right thyroid and above the brachiocephalic trunk PC	
Dilhara Karunaratne, et al. (29)	45/M	979	4.48	5000/N	>3	US: Inferior pole of the right thyroid MIBI: Uptake in inferior pole of the right thyroid	Inferior Right PC	Dead at 3-month
Yunhui Xin, et al. (30)	53/F	1900	3.72	2.5/1D	2.48	MIBI: High uptake in the mediastinum	Intrapericardial PC	
Wei Wang, et al. (31)	10/M	114	3.56			US: Right thyroid lobe MIBI: Uptake in inferior pole of the right thyroid	Inferior Right PC	

A blank form indicates that this information was not provided in the original case report.

X, Time of post-operative examination; PC, Parathyroid Carcinoma; PA, Parathyroid adenoma.

In our present case, the initial parathyroid US showed no abnormalities, while a subsequent US identified an abnormal parathyroid lesion. This discrepancy was attributed to the experiences of different operators. Moreover, dual-phase Tc-99m-MIBI parathyroid scintigraphy produced false-negative results in both early and delayed phases. The diagnosis of PC was ultimately confirmed intraoperatively and through histopathological examination. The false-negative Tc-99m-MIBI result may be explained by the presence of hemorrhage and necrosis within the parathyroid lesion, as observed during surgery.

## Conclusions

Regrettably, there is currently a lack of definitive preoperative diagnostic methods for parathyroid carcinoma (PC). The preferred diagnostic approach remains the combination of ultrasound and Tc-99m-MIBI SPECT/CT imaging. When severe hypercalcemia and parathyroid hormone (PTH) levels are 3-10 times higher than normal, PC should be considered. 18F-FDG PET/CT imaging is considered a sensitive and effective method for the initial staging of parathyroid carcinoma, tumor recurrence, assessment of residual lesions after treatment, and detection of distant metastases. 18F-choline PET/CT also holds significant value in localizing parathyroid carcinoma and seeking out metastatic lesions. A combined diagnostic approach, including imaging and serum biomarkers, can improve the preoperative diagnostic rate of PC.

In the future, we hope to develop specific targets for biomarkers in parathyroid tissue to facilitate early diagnosis and localization of PC and distant metastases. Such advancements would greatly enhance our understanding and treatment of parathyroid carcinoma, providing patients with more accurate diagnoses and more effective treatment plans.

## Data Availability

The original contributions presented in the study are included in the article/supplementary material. Further inquiries can be directed to the corresponding authors.
